# The association between fear of falling and smoothness of lower trunk oscillation in gait varies according to gait speed in community-dwelling older adults

**DOI:** 10.1186/s12984-016-0211-0

**Published:** 2017-01-19

**Authors:** Tsuyoshi Asai, Shogo Misu, Ryuichi Sawa, Takehiko Doi, Minoru Yamada

**Affiliations:** 1 Department of Physical Therapy, Faculty of Rehabilitation, Kobe Gakuin University, 518 Ikawadanicho, Arise, Nishi-ku, Kobe, Hyogo 651-2180 Japan; 2 Kobe City Hospital Organization, Kobe City Medical Center West Hospital, 1-2-4 Nagata-ku, Kobe, Hyogo 653-0013 Japan; 3 Department of Physical Therapy, School of Health Sciences at Narita International University of Health and Welfare 4-3, Kōzunomori, Narita-shi, Chiba-ken 286-8686 Japan; 4 Department of Health and Medical Care Center for Development of Advanced Medicine for Dementia, Section for Health Promotion, National Center for Geriatrics and Gerontology, 35 Gengo, Morioka, Obu, Aichi 474-8511 Japan; 5 Graduate School of Comprehensive Human Sciences, University of Tsukuba, 3-29-1 Otsuka, Bunkyo-ku, Tokyo, 112-0012 Japan

**Keywords:** Fear of falling, Gait, Accelerometer, Community-dwelling older adults

## Abstract

**Background:**

Fear of falling (FoF) is common in community-dwelling older adults. FoF and increased walking speed are associated with lower trunk oscillation during gait in older adults. We hypothesized that older adults with FoF would struggle to walk safely when instructed to walk faster than usual.

**Methods:**

Participants included 260 community-dwelling older adults aged over 65 years (mean age = 71.9 ± 3.9 years) who were able to walk independently without an assistive device. Participants were instructed to walk along a 15-m smooth horizontal walkway at self-selected normal and fast gait speeds. During the middle 10 m of the walk, oscillation of the lower trunk and stride times were measured with two accelerometers. We examined associations between gait variables, including harmonic ratio (HR) in vertical, mediolateral (HR-ML) and anteroposterior (HR-AP) directions as indicators of smoothness of lower trunk oscillation, as well as stride time variability (STV) and FoF.

**Results:**

Gait-speed- and STV- adjusted models showed that FoF was significantly associated with HR-ML in the normal-gait condition (HR-ML: β = - .135, *p* = .040), while FoF was significantly associated with HR-AP in the fast-gait condition (HR-AP: β = - .154, *p* = .017).

**Conclusions:**

FoF-related changes in gait vary with gait speed. In older adults with FoF, lower trunk oscillation was less smooth in the lateral direction when they walked at their usual pace. In addition, lower trunk oscillation was also less smooth in the direction of travel when they walked at a faster pace than their usual walking speed.

## Background

Fear of falling (FoF) is common among older adults, exhibited as a lack of self-confidence in performing normal activities without falling [1]. The prevalence of FoF increases with age, ranging from 21 to 85% in community-dwelling older adults [2]. FoF is an important risk factor for falling [1, 3] and for limitations in activities of daily living [4]. Additionally, FoF is associated with psychological problems [5] and poor physical performance [6, 7]. FoF-related issues have a serious impact on health and quality of life for older people. Thus, FoF is an important community health issue to address [8].

Unimpaired gait is an important factor influencing older adults’ ability to live independently in the community. In normal gait, trunk stability contributes, in part, to successful locomotion. During walking, the control of trunk oscillation is prioritized and the trunk plays an important role in providing a stable platform for vision and head control [9]. Importantly, some reports have shown that FoF is associated with the control of trunk oscillation while walking. Specifically, it has been reported that the acceleration waveforms of the trunk are less smooth and the trunk has greater amplitude in dual-task conditions among older adults with FoF, compared with those without FoF [10, 11]. Such gait changes contribute to the risk of falling among older adults [12, 13]. Thus, examining the association between FoF and the control of trunk oscillation during walking is of clinical importance.

Gait speed is one of the essential factors influencing the control of trunk oscillation. Several studies have investigated the relationship between gait speed and the control of trunk oscillation, revealing that walking faster than one’s normal speed affects trunk control [14, 15]. There are many occasions in daily life in which community-dwelling older adults are required to walk faster than normal (e.g., crossing roads). Therefore, physical assessment of older adults should include measurements of gait in various gait-speed conditions, especially in fast-gait conditions. However, previous studies have only observed FoF-related gait changes when participants were instructed to walk at their normal walking pace [10, 11, 16–18]. To our knowledge, no studies have investigated FoF-related gait changes when participants are instructed to walk faster than their normal pace.

Therefore, the first objective of this study was to examine the association between FoF and the control of lower trunk oscillation when older adults were instructed to walk in two conditions: at their normal pace, and at faster than their normal pace. Understanding differences in the association between smoothness of lower trunk oscillation and FoF in each gait speed condition would provide clinically useful information. We used harmonic ratio (HR) to assess the control of lower trunk oscillation. HR has been widely used to assess acceleration of the lower trunk while walking, providing a measure of the smoothness of waveforms of acceleration.

An experience of falling is a known cause of FoF, but FoF is also prevalent in older adults without a fall history [2]. Fall history and FoF are both associated with gait; however, a limited number of studies have investigated whether a fall history contributes to the association between FoF and gait parameters, and have reported mixed results [10, 16–18]. Moreover, to our knowledge, no previous study has examined whether fall history confounds the relationship between FoF and the smoothness of lower trunk oscillation while walking. Determining whether FoF contributes to the smoothness of lower trunk oscillation in gait, irrespective of fall history, would provide useful insight for understanding the phenomenon of FoF in older adults, particularly regarding its relationship to gait. Thus, the second objective of this study was to examine whether the association between FoF and the smoothness of lower trunk oscillation was altered by fall history in a gait-speed-dependent way.

## Methods

### Participants

Between April 2011 and October 2013, 330 community-dwelling older adults were recruited through a community organization for older people. Eligibility criteria for this study included being over 65 and under 80 years of age, and the ability to walk independently without an assistive device (*n* = 296), because the effects of aging on gait become significantly stronger after the age of 80 years [19], and walking with an assistive device significantly changes gait patterns. Participants were excluded if they had a self-reported history of neuromuscular disease that affected gait (e.g., stroke or Parkinson’s disease, *n* = 0), or cognitive impairment (presenting with either a Rapid Dementia Screening Test score < 7 [20] or a Mini Mental State Examination score < 24 [21], *n* = 31), assessed by a trained physical therapist. In addition, participants who did not complete the assessment of FoF and gait measurement were excluded (FoF, *n* = 3; gait measurement, *n* = 2). The final analyzed sample included 260 participants. Background characteristics were assessed using a questionnaire that included questions on the following: age, sex, medical conditions (musculoskeletal disease, hypertension, heart disease, diabetes mellitus [yes/no]), the number of medications used, and fall history in the previous year. Additionally, FoF was assessed using the question “Are you afraid of falling (yes/no)?” This format has been reported to have a high test–retest reliability and has the advantage of being straightforward and allowing for the easy generation of prevalence estimates [22, 23]. Based on the answer to this question, the sample was divided into older adults with FoF (FoF-group) and those without FoF (Non-FoF group). The anthropometric index (height and weight) was obtained in a physical examination. Physical function was examined using the Five-Chair-Stand test (5CS) [24], which assessed lower-extremity power, and the Timed Up and Go test (TUG), which assessed basic mobility function [25]. Procedures for the 5CS and TUG have been described previously [24, 25]. This study was carried out in accordance with the principles of the Declaration of Helsinki. The Research Ethics Committee of Kobe Gakuin University approved the study (Approval No. HEB100806-1). Informed consent was obtained from all participants prior to participation.

### Gait analysis

Participants were asked to walk along a 15-m smooth horizontal walkway while wearing appropriately sized shoes, which were checked beforehand. The middle 10-m section of the walkway was marked off by two lines, positioned 2.5 m from each end, to allow space and time for acceleration and deceleration. After familiarization with the task and apparatus, the participants were instructed to walk at two self-selected speeds using the following instructions: (i) walk at your normal speed (normal-gait condition); and (ii) walk as fast as you can, but do not run (fast-gait condition). The measurement of each gait-speed condition was conducted once, in order, with the normal-gait condition followed by the fast-gait condition. Time taken to walk the middle 10 m was measured with a stopwatch, and gait speed was expressed in meters per second.

Trunk and heel acceleration during gait were measured using two wireless miniature sensor units that contained an accelerometer (MVP-RF8; MicroStone, Nagano, Japan). One sensor unit was fixed to a belt at the level of the L3 spinous process and the other was attached to the posterior surface of the right heel with surgical tape; thus, acceleration could be measured without restricting the subject’s movement. Trunk linear accelerations were measured in the vertical (VT), anteroposterior (AP), and mediolateral (ML) directions while subjects walked along a walkway. All signals were sampled at 200 Hz and synchronously wirelessly transferred to a personal computer via a Bluetooth personal area network.

Signal processing was performed with commercially available software (MATLAB, Release 2014b; The MathWorks Japan, Tokyo, Japan). Before the analyses were performed, all acceleration data were low-pass filtered with a cutoff frequency of 20 Hz. On the basis of pilot testing, heel-contact events were identified as vertical acceleration peaks of the heel. Stride time variability (STV) was used as the index of gait variability. STV (%) = (standard deviation of stride time/mean stride time) × 100 [26].

The harmonic ratio (HR) was used to evaluate the control of trunk oscillation, indicating walking smoothness during gait [14, 15, 27]. The validity of this measure has been confirmed in younger and older adults, and lower HR has been reported to be associated with risk of falling and functional impairment [27–29]. The mathematical derivation of HR was based on the detailed description provided by Menz et al. [14]. Briefly, the acceleration signals at the trunk of each stride were broken down into individual sinusoidal waveforms using digital Fourier transform with a rectangular window function, and 20 harmonics were calculated based on each stride time. HRs in the VT and AP directions were calculated as the sum of the amplitudes of the first 10 *even* harmonics divided by the sum of the amplitudes of the first 10 *odd* harmonics, because among acceleration signals in VT and AP directions, a stable smooth gait pattern consists of acceleration patterns that repeat in multiples of two during a single stride. However, HRs in the ML direction were calculated as the sum of the amplitudes of the *odd* harmonics divided by the sum of the amplitudes of the *even* harmonics, because acceleration signals in the ML direction were only repeated once for any given stride. HRs per stride were determined and averaged across a steady walk, resulting in a mean HR for each direction of motion. The equations of HR in VT and AP directions, and in the ML direction, are shown below:

HR in VT and AP directions

$$ \mathrm{H}\mathrm{R}={\displaystyle \sum }Amplitudes\ ofeven\ harmonics/{\displaystyle \sum }Amplitudes\ of\ odd\ harmonics $$

HR in the ML direction

$$ \mathrm{H}\mathrm{R} = {\displaystyle \sum } Amplitudes\  of\  odd\  harmonics/{\displaystyle \sum } Amplitudes\  of\  even\  harmonics $$

Higher HR values indicate greater walking smoothness, and lower values indicate reduced smoothness [14].

### Statistical analyses

The background characteristics and gait variables were compared between the Non-FoF and FoF groups using unpaired *t*-tests or likelihood ratio tests. For gait variables, the effect sizes were calculated. Then, for each gait-speed condition (normal-gait condition or fast-gait condition), general linear regression models were used to investigate the association between FoF and gait variables (STV, HR-VT, HR-ML, and HR-AP), adjusting for covariates. In the first model, we adjusted for age, sex, height, weight, medical condition (which was found to be significantly different by bivariate analysis), and gait speed, because acceleration-based gait indices are reported to be strongly affected by gait speed (Model 1) [27]. Second, STV was included as another covariate, except in the model for STV (Model 2). Finally, fall history was included as another covariate (Model 3). The level of statistical significance for all analyses was set at *p* < .05. All statistical analyses were performed using commercially available software (JMP12.0; SAS Institute Japan, Tokyo, Japan).

## Results

The participants’ characteristics are summarized in Table 1. There were 202 participants (78%) in the Non-FoF group and 58 (22%) in the FoF group. Although there were no significant age differences between the two groups, the ratios of men, height, and weight were significantly higher in the Non-FoF group compared with the FoF group. The completion times for the TUG test were significantly shorter in the Non-FoF group than in the FoF group. The ratio of musculoskeletal disease was significantly higher in the FoF group than the Non-FoF group.

**Table 1:** Subject characteristics for the Non-FoF and FoF groups

Characteristics	All subjects *n* = 260	Non-FoF group *n* = 202	FoF group *n* = 58	*P* value
Age, years	71.9 ± 3.9	71.6 ± 3.8	72.7 ± 3.9	0.057
Sex (male), %	44	50	22	<0.001
Height, cm	156.2 ± 8.4	157.2 ± 8.5	152.9 ± 7.5	<0.001
Weight, kg	57.4 ± 9.8	58.1 ± 9.9	55.0 ± 8.9	0.031
Fall history (faller), %	18	16	26	0.091
TUG, s	6.6 ± 1.2	6.4 ± 1.0	7.3 ± 1.4	<0.001
5CS, s	8.8 ± 2.8	8.7 ± 2.8	9.2 ± 2.5	0.161
Medical conditions:				
Musculoskeletal disease, %	13	9	25	0.003
Hypertension, %	43	45	38	0.389
Heart disease, %	11	11	11	0.957
Diabetes mellitus, %	12	12	9	0.515
Number of medications used	2.1 ± 1.9	2.0 ± 2.0	2.3 ± 1.8	0.454

Values are means ± standard deviation or percentages. *P* values were calculated using unpaired *t* tests or likelihood ratio tests

*FoF* Fear of falling, *TUG* Timed Up and Go test, *5CS* Five Chair Stand test

**Fig. 1:**
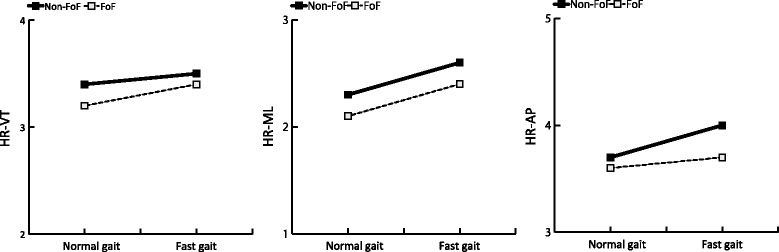
Harmonic ratio in vertical, mediolateral, and anteroposterior directions in normal- and fast-gait speed conditions. FoF: Fear of Falling, HR: Harmonic Ratio, VT: Vertical, ML: Mediolateral, AP: Anteroposterior

The results of the bivariate analysis of gait variables are shown in Table 2 and Fig. 1. Walking speed in both the normal- and fast-gait conditions was significantly slower in the FoF group compared with the Non-FoF group. The FoF group exhibited larger STV values in the normal-gait condition compared with the Non-FoF group. The effect sizes for gait speed in the normal gait and fast gait conditions were 0.5 (medium effect) and 1.0 (large effect), respectively. The STV effect size in the normal gait condition was 0.42 (small-medium effect). Other gait variables were not significantly different between the groups in either the normal- or fast-gait conditions.

**Table 2:** Comparison of gait variables at two gait speed conditions in the Non-FoF and FoF groups

Gait variables	Non-FoF group, *n* = 202		FoF group, *n* = 58	
	Normal gait	Fast gait	Normal gait	Fast gait
Walking speed, m/s	1.4 ± 0.2	1.8 ± 0.2**	1.3 ± 0.2^‡^	1.6 ± 0.2**^‡^
STV, %	1.9 ± 0.9	2.1 ± 1.1	2.3 ± 1.1^†^	1.9 ± 0.9^*^
HR-VT	3.4 ± 0.9	3.5 ± 0.9	3.2 ± 0.9	3.4 ± 1.1
HR-ML	2.3 ± 0.7	2.6 ± 0.8**	2.1 ± 0.7	2.4 ± 0.7*
HR-AP	3.7 ± 1.1	4.0 ± 1.1**	3.6 ± 1.0	3.7 ± 1.2

Values are means ± standard deviation. Normal gait is a gait condition in which subjects were asked to walk at a self-selected preferred gait speed. Fast gait is a gait condition in which subjects were asked to walk at a self-selected fast-gait speed

*FoF* Fear of Falling, *STV* Stride Time Variability, *HR* Harmonic Ratio, *VT* Vertical, *ML* Mediolateral, *AP* Anteroposterior

^†^Gait variable was different between two groups at *P* < 0.05

^‡^Gait variable was different between two groups at *P* < 0.01

*Gait variable was different between two speed conditions at *P* < 0.05

**Gait variable was different between two speed conditions at *P* < 0.01

The standard beta coefficients from the general linear regression analyses investigating the association between FoF and gait variables in the normal and fast-gait conditions are shown in Table 3. In Model 1, which adjusted for demographic factors (age, sex, height, weight, musculoskeletal disease) and gait speed, HR-ML and STV were significantly associated with FoF in the normal-gait condition, and HR-AP was associated with FoF in the fast-gait condition (normal-gait condition, HR-ML: adjusted R-squared = 0.093, standard β = -0.145, *P* = 0.026, STV: adjusted R-squared = 0.017, standard β = 0.178, *P* = 0.009, fast-gait condition, HR-AP: adjusted R-squared = 0.049, standard β = -0.143, *P* = 0.032). In Model 2, which included STV as another covariate, HR-ML was significantly associated with FoF in the normal-gait condition (normal-gait condition, HR-ML: adjusted R-squared = 0.092, standard β = -0.135, *P* = 0.040). HR-AP was significantly associated with FoF in the fast-gait condition, and the adjusted R-squared value increased (HR-AP: adjusted R-squared = 0.110, standard β = -0.154, *P* = 0.017). In Model 3, which included fall history as another covariate, these associations were not attenuated further (normal-gait condition, HR-ML: adjusted R-squared = 0.089, standard β = -0.135, *P* = 0.041, STV: adjusted R-squared = 0.014, standard β = 0.179, *P* = 0.009, fast-gait condition, HR-AP: adjusted R-squared = 0.107, β = -0.154, *P* = 0.018).

**Table 3:** Association between harmonic ratios and fear of falling and in normal and fast-gait conditions

Gait speed condition	Model Independent variables	Dependent variables			
		HR-VT	HR-ML	HR-AP	STV
		Standard β (*P* value)	Standard β (*P* value)	Standard β (*P* value)	Standard β (*P* value)
Normal gait	Model 1				
	FoF	-0.083 (0.210)	-0.145 (0.026)	-0.088 (0.170)	0.178 (0.009)
	Gait speed	0.201 (0.002)	0.095 (0.136)	0.231 (<0.001)	-0.015 (0.819)
	Adjusted R^2^	0.051	0.093	0.107	0.017
	Model 2				
	FoF	-0.043 (0.509)	-0.135 (0.040)	-0.050 (0.435)	
	Gait speed	0.198 (0.002)	0.094 (0.140)	0.227 (<0.001)	
	STV	-0.223 (<0.001)	-0.054 (0.383)	-0.217 (<0.001)	
	Adjusted R^2^	0.097	0.092	0.150	
	Model 3				
	FoF	-0.044 (0.500)	-0.135 (0.041)	-0.050 (0.431)	0.179 (0.009)
	Gait speed	0.202 (0.002)	0.093 (0.151)	0.229 (<0.001)	-0.019 (0.779)
	STV	-0.223 (<0.001)	-0.054 (0.380)	-0.217 (<0.001)	-------
	Fall history	-0.030 (0.633)	0.014 (0.823)	-0.167 (0.786)	0.029 (0.656)
	Adjusted R^2^	0.097	0.089	0.146	0.014
Fast gait	Model 1				
	FoF	-0.079 (0.239)	-0.107 (0.101)	-0.143 (0.032)	-0.043 (0.519)
	Gait speed	-0.066 (0.349)	0.209 (0.002)	0.045 (0.518)	0.155 (0.027)
	Adjusted R^2^	0.032	0.086	0.049	0.043
	Model 2				
	FoF	-0.091 (0.163)	-0.114 (0.080)	-0.154 (0.017)	
	Gait speed	-0.024 (0.722)	0.232 (<0.001)	0.085 (0.210)	
	STV	-0.268 (<0.001)	-0.149 (0.017)	-0.260 (<0.001)	
	Adjusted R^2^	0.097	0.104	0.110	
	Model 3				
	FoF	-0.091 (0.162)	-0.114 (0.080)	-0.154 (0.018)	-0.043 (0.517)
	Gait speed	-0.023 (0.743)	0.232 (<0.001)	0.081 (0.237)	0.156 (0.028)
	STV	-0.268 (<0.001)	-0.149 (0.018)	-0.260 (<0.001)	-------
	Fall history	-0.014 (0.822)	-0.005 (0.941)	0.032 (0.606)	-0.009 (0.888)
	Adjusted R^2^	0.094	0.100	0.107	0.039

All models were adjusted for age, sex, height, weight, musculoskeletal disease using a general linear regression model. Normal gait is a gait condition in which subjects were asked to walk at a self-selected preferred gait speed. Fast gait is a gait condition in which subjects were asked to walk at a self-selected fast-gait speed

*FoF* Fear of Falling, *STV* Stride Time Variability, *HR* Harmonic Ratio, *VT* Vertical, *ML* Mediolateral, *AP* Anteroposterior

## Discussion

The current results demonstrated that the association between FoF and HRs changed when people were requested to walk faster than their usual walking pace. HR-ML was significantly associated with FoF in the normal-gait condition, while HR-AP was significantly associated with FoF in the fast-gait condition. These results indicate that FoF-related changes in gait may vary according to gait-speed conditions. Our findings suggest that older adults with FoF show less smooth lower trunk motion, particularly in the lateral direction, when they walk at a normal-gait speed, and show less smooth lower trunk motion in the direction of travel when they walk at a pace that is faster than their usual speed. To the best of our knowledge, the present study is the first to demonstrate a relationship between FoF and trunk control during gait in fast-gait walking.

The Non-FoF group showed significantly greater HR-AP values in the fast-gait condition compared with the normal-gait condition. This result is consistent with previous studies reporting that high HR-AP values were maintained at a faster pace [30–32]. However, the FoF group showed no change in HR-AP between gait speed conditions. These results imply that the positive effect on HR-AP related to faster gait speed may be attenuated by FoF, resulting in an association between HR-AP and FoF only in the fast-gait condition. In addition, in the present study, the association between HR-AP and FoF was investigated adjusting for gait speed and STV using a multi-regression model, because STV was considered an important factor influencing anteroposterior trunk control [33]. The modeling results revealed that the association between FoF and HR-AP was strengthened after adjusting for STV (Model 1: FoF: standard β = -0.143, Model 2: FoF: standard β = -0.154). These findings indicate that FoF is associated with the smoothness of lower trunk motion in the direction of travel, independent of gait variability. However, further research will be necessary to test the effects of other potential confounding variables that may influence this association.

The FoF group showed significantly lower HR-ML values in the normal-gait condition. This result is consistent with other studies investigating trunk control at normal gait speed, which reported that lateral trunk control was highly prioritized compared with the other two directions [14, 34]. Moreover, previous dual-task studies conducted by our research team revealed that an additional task mainly affects lateral trunk control while walking [35, 36]. Together, these findings indicate that highly prioritized lateral trunk control may require the allocation of substantial attentional resources. Thus, FoF may reduce the amount of attentional resource allocated to lateral trunk control, potentially affecting lateral lower trunk motion. However, the dual-task protocol was not adopted in the present study, and further studies are required to clarify this possibility. In contrast, FoF was not associated with HR-ML in the fast gait condition. This phenomenon can be partially explained by the displacement of the center of mass during fast walking. One previous study reported that the ML center of mass displacement became smaller as walking speed increased in young adults, observing an approximate 13% decrease when walking speed changed from 1.2 m/sec to 1.6 m/sec [37]. Such changes during fast gait may enhance the smoothness of acceleration waveforms. This positive effect on trunk motion may weaken the association between FoF and HR-ML. Additionally, step width and step width variability, which affect frontal plane trunk kinematics [38], vary during fast gait speed, and may be another factor influencing the current results [39]. To the best of our knowledge, no studies have investigated how these gait parameters contribute to lateral trunk control. Thus, further research is needed to clarify these issues.

The current results revealed that older adults with FoF walked slower than those without FoF. In addition, greater STV was observed in older adults with FoF than those without FoF in the normal-gait condition. Some previous studies have suggested that FoF causes people to walk more cautiously [16, 40]. This cautious gait pattern is characterized by a high degree of gait variability in spite of slow gait speed, reduced stride length and widening of the base of support, and is classified as a non-specific high-level gait control disorder [41–43]. Thus, the current results confirm the link between FoF and cautious gait pattern. Moreover, STV was associated with FoF after adjusting for gait speed. This result indicates that gait fluctuations may remain after removing the effect of slower gait speed, which is also a common feature of cautious gait. Conversely, STV was not significantly associated with FoF after controlling for gait speed in the fast-gait condition. Several previous studies are in accord with these findings [44, 45], including a report that increasing gait speed improves interlimb coupling in younger people [44]. In addition, variability of electromyography (EMG) patterns in lower limbs have been found to decrease with increasing gait speed [45]. Thus, consistency of lower limb movement related to fast gait may weaken the contribution of FoF to step fluctuation.

In both the normal- and fast-gait conditions, the associations between gait parameters and FoF were not changed in the multiple regression model after controlling for fall history. Thus, these results indicate that FoF-related changes of trunk control in gait may occur irrespective of fall history. Our findings are consistent with those of other studies reporting significant associations between FoF and gait parameters adjusted for several potential confounders, including fall history [10, 17, 18]. However, the findings of another study by Ayoubi et al. are not consistent with our results, reporting that a combination of FoF with fall history was significantly associated with increased STV only if walking speed was above 1.14 m/s [16]. Although the gait speed in our study sample was much faster than this gait speed (mean gait speed 1.4 m/s in the normal-gait condition), and other parameters were similar to Ayoubi et al.’s sample, we found that fall history did not affect the association between FoF and gait parameters. This discrepancy provides some insight regarding fall history and its relationship with the association between FoF and gait parameters; considering the difference in gait speed between the two studies, the effect of fall history may vary depending on basic gait function. Indeed, our sample showed a relatively high level of gait function, and may be categorized as well-functioning older adults. Thus, for such older adults, fall history may not affect both FoF and gait function, but, for older adults with moderate gait function, fall history may play an important role in the association between FoF and gait. FoF is known to be affected by various factors, including mental and physical condition [2]. Exploration of the underlying causes of FoF is an important fall-related research issue, warranting further study.

In the field of geriatric rehabilitation, the current results provide clinically useful information for management of fall risk, because rehabilitation staff members are often required to provide postural support at the side of older adults during walking exercises. Our results revealed that older people with FoF tend to exhibit trunk movement fluctuations in the lateral direction when instructed to walk at their usual pace, and show alterations in trunk movement in the traveling direction when they are instructed to walk at a faster pace. This pattern indicates that staff members may be able to adjust their position to provide better postural support to older people with FoF, according to their walking speed. Fall risk management is a high priority in geriatric rehabilitation. The current results may provide valuable insight for improving the safety of walking exercise in older people.

The current study has several limitations that should be considered. First, the study did not include older adults with decreased physical function, such as frailty. Previous studies have found that older adults with decreased physical function experience FoF more often than well-functioning older adults [2, 4]. The association between FoF and gait in such individuals may differ from the present study sample, which exhibited intact physical function. As we did not include older adults with decreased physical function in the present study, we cannot generalize our results to populations with impaired mobility. Another limitation is that FoF was not assessed using other FoF assessment methods, such as the Fall Efficacy Scale [46]. FoF is often not acknowledged, and may be minimized by an individual. The Fall Efficacy Scale quantifies FoF and classifies individuals according to their level of FoF. Future studies should examine the differences between varying levels of FoF and postural control when older adults with FoF walk faster than usual. Finally, a high level of variance in each gait parameter cannot be accounted for by the independent variables that were used in the regression models in the current study. The R-squared values of HRs in a general linear regression model (Model 2), adjusted for common gait parameters, gait speed and STV, were 0.092 for HR-ML in normal gait and 0.11 for HR-AP in fast gait. These results indicate that the strength of association between fear of fall and tested-gait parameters was low. A systematic review reported that dizziness, depression, and self-rated health status are risk factors for the development of FoF [2]. Taken together with the findings of previous studies, the current results suggest that the association of FoF and gait parameters can be confounded by other risk factors of FoF. Such risk factors are diverse, and depend on the characteristics of the study sample. These factors were not examined in the present study, and should be clarified in future research.

## Conclusions

Overall, the current study revealed that FoF-related changes in gait varied with gait speed. In older adults with FoF, oscillation of the lower trunk was less smooth in the lateral direction when participants walked at their usual pace, and was less smooth in the direction of travel when participants walked faster than their usual pace.

## Abbreviations

5CS: Five-Chair-Stand test
AP: Anteroposterior
FoF: Fear of falling
HR: Harmonic ratio
ML: Mediolateral
STV: Stride time variability
TUG: Timed Up and Go test
VT: Vertical
